# Modulation of the Complement System by Human β-Defensin 2

**Published:** 2007-01-10

**Authors:** Satyanarayan Bhat, Yau-Hau Song, Carl Lawyer, Stephen M. Milner

**Affiliations:** a The Institute for Plastic and Reconstructive Surgery, Southern Illinois University School of Medicine, Springfield, IL; b Johns Hopkins University School of Medicine, Baltimore, MD

## Abstract

**Objective:** Human β-defensin (HBD) and the complement system are two important innate immune mechanisms active against a broad range of burn and wound pathogens. However, excessive or uncontrolled complement activation, following thermal injury, contributes to tissue damage. Previous studies from our laboratory suggested a decreased expression of HBD-2 in burn wounds and its absence in burn blister fluid. Prior studies have demonstrated that human neutrophil peptide can bind to the C1q component of the complement system and prevent complement activation. The objective of this study was to determine whether HBD-1 and HBD-2 can also bind to the C1q component and modulate complement activity. **Methods:** The binding efficiency of HBD-1 and HBD-2 to the C1q component was determined by utilizing dot blot hybridization. The effect of HBD-2 on the activation of the complement system by the classical and alternative pathways was determined by CH50 and AP50 assays. In addition, the ability of HBD-1 and HBD-2 to inhibit C1q activity was predicted by a comparison with known C1q inhibitor peptide 2J in a DNAStar computer modeling study. **Results:** C1q binding to HBD-2 was strong, whereas C1q binding to HBD-1 was weak. HBD-2 inhibits the classical pathway significantly without affecting the alternative pathway. In addition, a computer modeling study also revealed structural homology of HBD-2 with known C1q inhibitory sequences of HBD-2. **Conclusion:** HBD-2 inhibits the classical pathway. The replacement of missing defensin, a natural inhibitor of the complement system, may have a dual protective role not only as an antimicrobial agent but also in providing protection against uncontrolled activation of the complement system.

Burn wound sepsis is a common and often fatal sequel in patients suffering from major thermal injury.^1^ Severely burned skin ceases to perform its natural protective role and surrenders itself as a nidus and portal for bacterial invasion. Multiple organ failure syndrome remains a problem in patients with extensive deep burns. The activation of a proinflammatory cascade after burn injury appears to be important in the development of subsequent immune dysfunction, susceptibility to sepsis, and multiple organ failure.^1^–^5^ Most therapeutic strategies directed specifically toward neutralizing inflammatory mediators or cytokines to control sepsis have failed in clinical trials, and the treatment of established organ failure is usually not successful.^2^ Despite various current topical treatment regimens designed to eradicate the bacterial load within the burn wound, sepsis remains the leading cause of death in burn units around the world.^1^–^3^

Thermal injury is associated with immune suppression, which includes both innate and adaptive immune systems.^4^–^6^ Innate immunity is largely conferred by the complement system, leukocytes, and recently characterized antimicrobial peptides, which include defensins and cathelicidins.^7^–^9^ A problem arises if the innate immune mechanisms are overactive, a feature observed in burns.^8^

Defensins, a large family of broad-spectrum antimicrobial peptides, are expressed at the cell surface and function as a biochemical barrier against microbial infection by inhibiting colonization of a wide range of pathogenic microorganisms, including burn wound pathogens.^7^,^9^,^10^ In humans, defensins are classified into 2 families designated α and β based on distinctive, although similar, tri-disulfide linkages. Six α-defensins—human neutrophil peptides (HNP), HNP-1 to HNP-4, human defensin 5 (HD-5), and HD-6—and 4 β-defensins—human β-defensins, HBD-1 to HBD-4—have been identified in humans. The defensins are also implicated in potentiating innate and adaptive immunity; have been shown to induce histamine release by mast cells,^10^ chemoattract neutrophils, macrophages, monocytes, T cells and B cells as well as dendritic cells; and alter antibody production.^11^,^12^

The activation of the complement system is an important first line of defense against microbial infection.^8^,^13^,^14^ Its principal role is the recruitment of inflammatory cells and the killing or opsonization of microorganisms. The complement system consists of more than 30 serum and cellular proteins. Activation of the system occurs by 3 biochemical pathways, the classical, the alternative, and the mannin-binding lectin (MBL) (Fig 1). The *classical pathway* is initiated when antibodies bind to antigens on the surface of the microbe. The subsequent binding of this antibody complex to C1, the first component of the complement system, splits C1 into subcomponents C1q, C1r, and C1s. Released C1r and C1s are proteases that activate the cascade. The *MBL pathway* is triggered by the binding of MBL in serum to polysaccharides on the surface of the microbe and activation of MBL-associated serine proteases. The *alternative pathway* is initiated by deposition of the spontaneously generated C3b component on bacterial surfaces. All 3 steps terminate in a final common pathway, ultimately leading to the formation of the membrane attack complex that lyse microorganisms. The C3b fragment deposited on the microbial surface can serve as an opsonin to promote phagocytosis.^9^ Activation of the complement system is well controlled internally. Its overactivation is mainly prevented by a number of naturally occurring inhibitors, such as the inhibitor of C1, C1-Inh, which inhibits the serine proteases C1r and C1s of the classical pathway.

It has been shown that human neutrophil defensins predominantly bind to the first component C1 and C1-Inh,^15^ suggesting that complex formation between defensins and C1 inhibit activation of the complement system and may also inhibit the cytolytic activity of defensins.^15^ In our previous studies we have shown that the expression of HBD-2 is reduced in burned skin and absent in burn blister fluid.^16^–^19^ The absence of HBD-2 may lead to decreased local antibacterial action and lack of inhibition of an overactive complement system, which might predispose the person to the development of sepsis and multi organ failure. The interaction between HBD and the complement system has not been previously reported. We have, therefore, studied the binding of HBD-1 and HBD-2 to C1q, to assess whether this interaction will affect the classical and alternative complement pathways. We have also compared the structural homology between HBD-2 and the known C1q-binding sequence of complement inhibitors using computer modeling.

## MATERIAL AND METHODS

### Dot blot hybridization

Full-length HBD-1 and HBD-2 peptides were synthesized at the Louisiana State University Medical Center, Core Laboratory. Peptides (20 mg/mL) were dissolved in 88% formic acid. The levels of binding of full-length HBD-1 and HBD-2 peptides to C1q were determined by dot blot hybridization. Four microlitres of peptide (HBD-1, HBD-2, or bovine serum albumin [BSA]) samples diluted with tris-buffered saline (TBS) to achieve required concentrations were blotted onto Trans blot transfer membrane (0.2 μm, Bio-Rad, Hercules, Calif). All incubations were at room temperature unless otherwise indicated. The membranes were rinsed in 20 mL of tris-buffered saline with Teween-20 (TBST), blocked with 20~mL of 5% nonfat dry milk in TBST for 1 hour, and rinsed briefly with TBST. All subsequent incubations contain TBST plus 1% nonfat dry milk. The membrane was incubated with human C1q (Quidel, San Diego, Calif; 10 μg/mL) for 3 hours. The membrane was then washed and incubated with rabbit antihuman C1q antibody (DAKO, Carpinteria, Calif) diluted 1:1000 in 1% nonfat dry milk in TBST. After washing, the membrane was incubated with goat antirabbit IgG, HRP (Sigma-Aldrich, St. Louis, Mo) at a dilution of 1:500 for 1 hour. The membrane was washed again and the signal was detected using the ECL system (Amersham Bioscience, Piscataway, NJ). The membrane was exposed to CH-screen for 45 minutes, and the image was quantified using the Phosphor Imager (GS-250, Bio-Rad, Hercules, Calif).

### Complement activity

The CH50 and AP50 hemolytic assays were adapted from the published procedure by Roos et al^20^ to determine activation of the complement system by the classical and alternative pathway, respectively, and to study the inhibitory properties of test peptides on complement activation. The CH50 hemolytic assay and AP50 hemolytic assay kits were purchased from Advanced Research Technologies (San Diego, Calif) and used as per manufacturer's instruction.

For hemolytic assays of classical pathway complement activation, sheep red blood cells were sensitized using rabbit antisheep red blood cell anti-bodycoated erythrocytes. For analysis of classical pathway activity, a hemolytic activity of the classical component pathway (CH50) test was performed, in which 1 × 10^7^ erythrocytes were incubated in the presence of normal human serum diluted in dextrose gelatin Veronal buffer (0.5 × VBS, 0.05% gelatin, 167 mM glucose, 0.15 mM CaCl_2_, 0.5 mM MgCl_2_; final volume 200~μL). Similarly, a C1q-dependent hemolysis test was performed using C1q-depleted human serum, diluted 1/75, and a limiting amount of purified C1q. For analysis of alternative pathway activity, a hemolytic activity of the alternative complement pathway (AP50) test was performed, in which 1 × 10^7^ rabbit erythrocytes were incubated with human serum diluted in dextrose gelatin Veronal buffer containing 10 mM MgEGTA. In all these assays, HBD-2 was tested by premixing appropriate concentrations of HBD-2 with the complement source before addition of the mixture to the erythrocytes, followed by 60-minute incubation at 37°C. After addition of 1.5 mL of phosphate buffered saline (PBS) and centrifugation, hemolysis was assessed by measuring the optical density (OD) at 414 nm. The lytic activity of condition *x* was expressed in *Z* values: *Z* = −ln{1 − [OD_414_ (*x*)− OD_414_ (0%)/(OD_414_ (100%) − OD_414_ (0%))]}, in which OD_414_ (0%) represents incubation of erythrocytes with buffer only. OD_414_ (100%) was assessed after addition of H_2_O. The amount of complement added was chosen such that the *Z* value in the absence of inhibitors was between 0.5 and 1.5. In general, serum was diluted 1/200 for a CH50 assay and 1/10 for an AP50 assay. The results are expressed as relative hemolytic activity and calculated as the ratio of the *Z* value in the presence of the inhibitor and the *Z* value in the absence of the inhibitor. In some experiments, the percentage lysis was determined relative to a reagent blank and 100% lysis, expressed as units/mL (*Z*) and converted to percentage inhibition.

### Comparison of sequence homology

Comparison of alfa helix display of consensus sequence between peptide 2J^21^ and HBD-2 and their overlap were carried out using Laser Gene software (DNAStar, www.dnastar.com).

### Statistical analysis

All data are expressed as the mean ± SEM of at least separate experiments unless otherwise stated in the figure legend. Statistical analysis was performed using 2-tailed Student *t* test for unpaired samples with unequal variances. Differences were considered statistically significant at *P* < .05.

## RESULTS

### HBD-2 binds strongly to C1q whereas HBD-1 binds poorly

**Figure 2:**
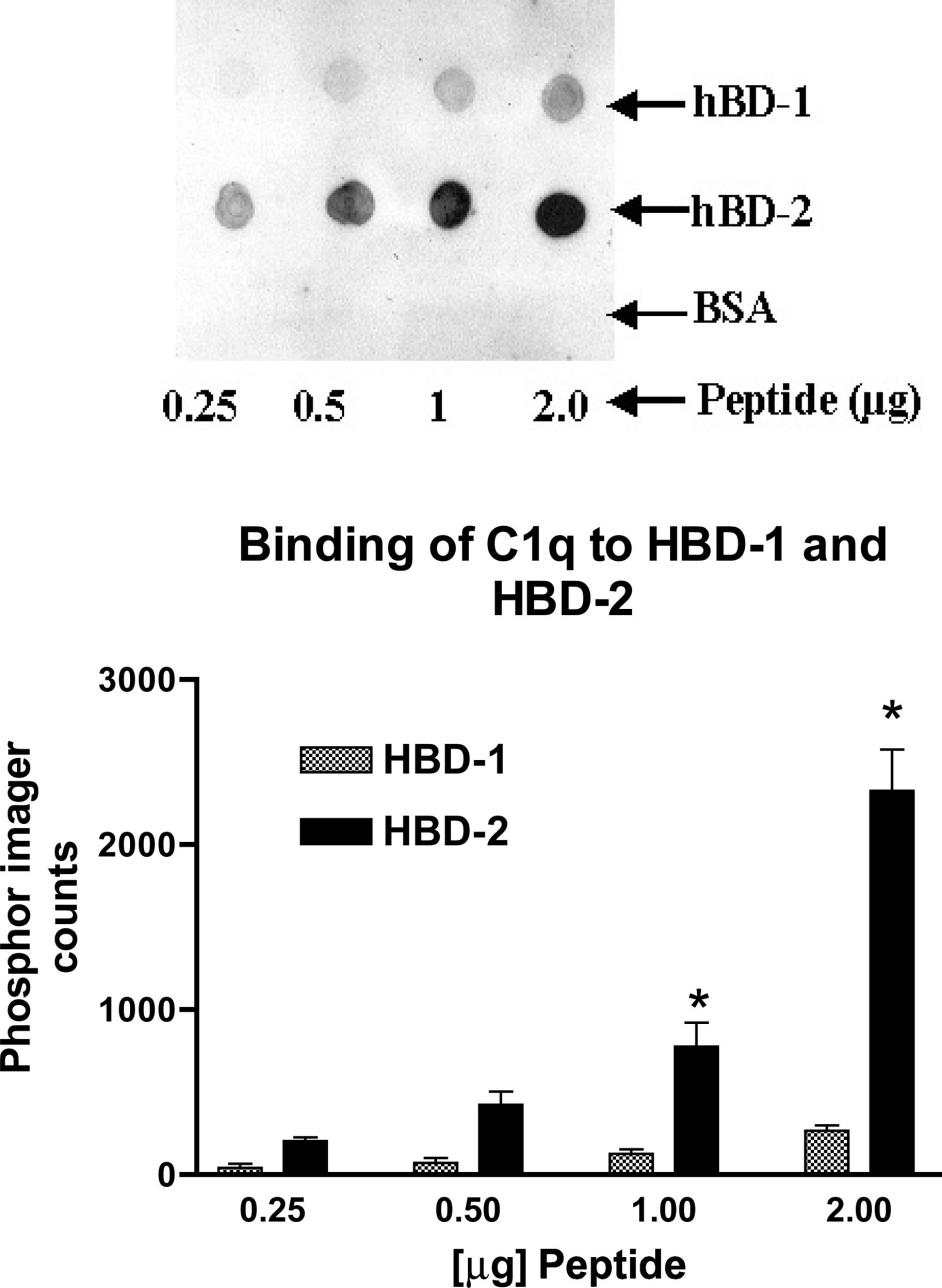
The dot blot hybridizations were carried out as described in the “Material and Methods” section. The quantified Phosphor Imager counts were plotted against different peptide concentrations. *Significance (*P* < .05).

Our data (Fig 2) show that HBD-2 binds strongly to C1q in a dose-dependent manner, whereas HBD-1 binds poorly. The quantified volume counts of dots in the phosphor imager are demonstrated in the bar chart in Figure 2. HBD-2 bound significant amounts of C1q even at a concentration of 0.25 μg, whereas HBD-1 did not show any detectable binding at this concentration. The BSA, used as a control protein to detect unspecific binding, did not show any C1q binding, indicating that the binding of C1q to HBD-2 is specific.

### HBD-2 inhibits the classical complement pathway without affecting the alternative pathway

**Figure 3:**
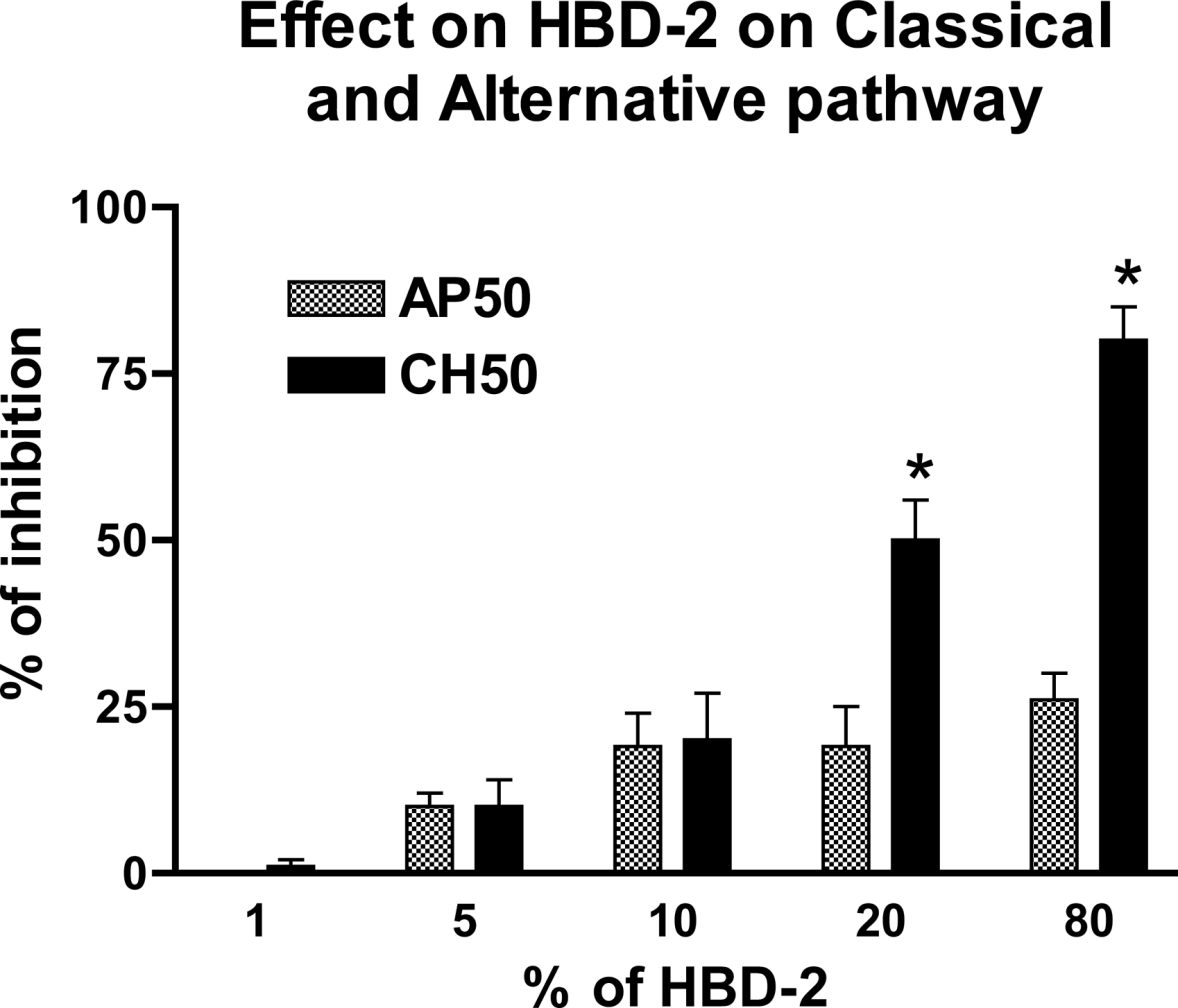
The effect of HBD-2 on hemolytic activity (CH50 and AP50) was determined as described in the “Material and Methods” section. The percentage of lysis was determined relative to a reagent blank and 100% lysis, expressed as units/mL (*Z*) and converted to percentage inhibition. *Significance (*P* < .05).

The effect of HBD-2 on classical and alternative pathways of complement activation was studied using hemolytic assays. Our data (Fig 3) show that binding of HBD-2 is accompanied by inhibition of the classical pathway of complement activation without affecting the alternative pathway.

### HBD-2 shows homology with known inhibitor of C1q activation

**Figure 4:**
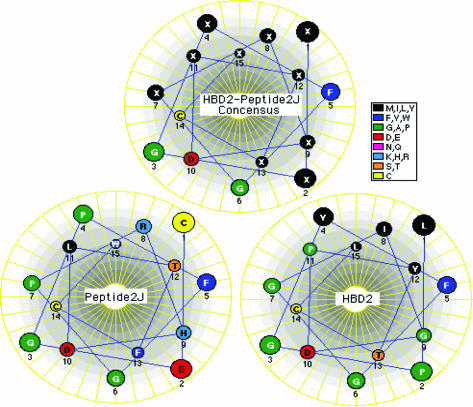
Alfa helix display of consensus sequence between peptide 2J and HBD-2 and their overlap using Laser Gene software.

Roos and coworkers^20^ screened 42 peptides for their ability to inhibit the function of C1q. These peptides were selected from phage-displayed peptide libraries on the basis of their C1q-binding ability, and it was found that peptide 2J was the most active C1q inhibitor. Peptide 2J [CEGPFGPRHDLTFCW] inhibited the hemolytic activity of C1q from human, chimpanzee, rhesus monkey, rat, and mouse with a similar dose-response relationship. Comparison of the homologous region (“Consensus”) [GXFGXXXDXXXC] between HBD-2 [LPGVFGGIGDPVTCL] and peptide 2J [CEGPFGPRHDLTFCW] may explain the observed inhibition of C1q by both HBD-2 and peptide 2J. This GXFGXXXDXXXC consensus is primarily on one side of an alpha helix (Fig 4) and is in the region of HBD-2 that contains the protease cleavage site where the signal peptide is removed by tissue metalloprotease (“matrilysin”) to produce active HBD-2. Thus, both peptide 2J and HBD-2 may be able to bind to the active site of C1q (globular head) and act as (protease) inhibitors to produce the observed inhibition of C1q activation.

## DISCUSSION

In this study, we have demonstrated that HBD-2 can bind to the C1q component of the complement system and inhibit activation of the classical pathway. A study from Van Den Berg and coworkers has shown that the binding of HNP-1 to C1q inhibited the activation of the classical pathway as evidenced by CH50 assay.^22^

Complement activation is a very important innate immune defense mechanism against invading microbes. Some activation is necessary for an efficient clearance of bacteria or their products, while excessive or uncontrolled activation can lead to inflammation and tissue injury, as seen with thermal injury.^23^ The C3a, C4a, and C5a peptides released during complement activation leads to increased inflammatory responses.^24^–^26^ Complement component C5a and membrane attack complex (MAC) have proinflamatory effects such as accumulation and stimulation of neutrophils and may increase the permeability of endothelial cells, mediated in part by histamine, and promote coagulation by inducing expression of tissue factor. C5a is able to induce or enhance the production of cytokines IL-1, TNF, and IL-6 by monocytes. Inhibition of complement activation is considered a major target for drug design and treatment of inflammation-related disorders.^20^,^21^,^27^–^30^ In a porcine model for thermal injury, C1-Inh was shown to have beneficial effects in the acute stage of thermal injury by reducing organ alterations, improving microcirculation, and largely preventing bacterial translocation in the gastrointestinal tract.^8^ Favorable effects of C1-Inh administration to patients with burns have been reported.

The therapeutic application of complement inhibitors, to prevent undesired effects of complement activation, is currently under development. The main culprit for tissue damage is the classical pathway and a number of inhibitors of this pathway have a proven efficacy in a number of diseases including burns and sepsis. This includes the use of C1-Inh and IgG.^8^,^13^ The soluble form of CR1 receptor and Compstatin, a synthetic peptide inhibitor of C3, have also been shown to prevent complement-related damage.^29^,^30^

C1q is considered one of the main targets identified for complement inhibition. Recently, Lauvrak and coworkers reported the sequences of 42 peptides that were selected from phage display libraries on the basis of their ability to bind to human C1q.^21^ These peptides were studied in detail for their anticomplement activity. The peptide 2J (15 amino acids) showed optimum C1q-binding properties. This peptide inhibits C1q from human, primate, and rodent origins and was proposed as a promising candidate for further development as a therapeutic C1q inhibitor.^20^ Using computer modeling we found that HBD-2 shares homology with peptide 2J. We therefore hypothesize that HBD-2 qualifies as a complement inhibitor.

In summary, we report binding of HBD-2 to the C1q component of the complement system and inhibition of its classical pathway. Computer modeling has revealed significant homology between HBD-2 and known complement inhibitor peptide 2J, predicting interaction between HBD-2 and C1q. To our knowledge, this is the first report of the interaction of human beta defensins and the complement system, findings that require a detailed study to explore the utility of such interaction in thermal injury and sepsis.

## Figures and Tables

**Figure 1:**
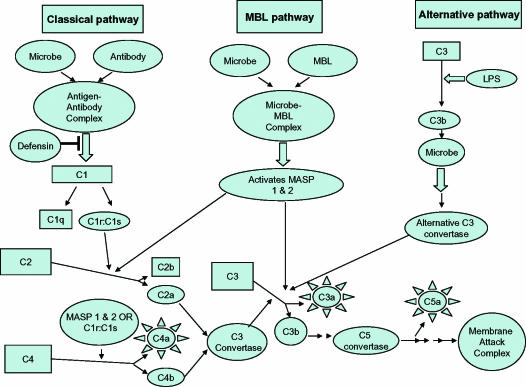
The complement system consists of about 30 proteins that can be activated by 3 different cascades. The classical pathway is initiated when antigen-antibody complexes bind to the C1q component of C1, resulting in release of C1s and C1r, which act as protease to cleave C2 and C4. The mannin-binding lectin (MBL) pathway is triggered by activation of MBL-associated serine proteinases (MASP-1 and MASP-2) following the binding of polysaccharides on microbes to MBL. MASP-1 and MASP-2 convert C2 and C4. The alternative pathway is triggered by the deposition of spontaneously generated C3b on microbes, leading to the generation of alterative C3 convertase. The common final step is the activation of C5 by C5 convertases, which leads to the formation of the membrane attack complex that finally inserts into target cell membranes and causes cell lysis. The peptides C3a, C4a, and C5a mediate several reactions in the inflammatory response that could cause tissue injury when complement activation becomes uncontrolled.
